# Adherence to Antihypertensive Treatment and Associated Factors in Central Ethiopia

**DOI:** 10.1155/2020/9540810

**Published:** 2020-10-19

**Authors:** Daniel G/Tsadik, Yemane Berhane, Alemayehu Worku

**Affiliations:** ^1^Department of Nursing, School of Health Sciences, Arsi University, Asella, Ethiopia; ^2^Addis Continental Institute of Public Health, Addis Ababa, Ethiopia; ^3^School of Public Health, Addis Ababa University, Addis Ababa, Ethiopia

## Abstract

**Background:**

Adherence to treatment is a primary determinant of treatment success. Nonadherence attenuates clinical benefits to the recipients of the treatment. However, monitoring adherence to long treatment regimens is not given due emphasis in low-income contexts. This study aimed to assess adherence to antihypertensive treatment and factors associated with it in Central Ethiopia.

**Method:**

This is an institution-based cross-sectional study conducted in 4 public hospitals in Central Ethiopia from December 4, 2016, to September 25, 2017. The Morisky Medication Adherence Scale (MMAS-8) was used to assess the level of adherence. The Revised Illness Perception Questionnaire (IPQ-R) was used to assess illness perception. The MMAS-8 score ranges from 0 to 8, a score of 8 reflects high adherence, 6 to 7 medium adherence, and <6 low adherence.

**Results:**

A total of 989 hypertensive patients participated in the study, of which 36.0% were assessed to have high adherence, 31.7% medium adherence, and 32.3% low adherence. We found that treatment adherence was significantly and positively associated with having family support (AOR: 1.65; 95% CI = 1.23, 2.22), high perception about consequences of hypertension (AOR: 1.51; 95% CI = 1.17, 1.95), and high perception about the severity of the disease (AOR: 1.42; 95% CI = 1.09, 1.86).

**Conclusion:**

The treatment adherence to antihypertensive medications is low in Central Ethiopia. Engaging family members in the treatment plan and improving patients' understanding of the illness are critical in achieving high adherence to medication in this context.

## 1. Introduction

Hypertension (HTN) is one of the major public health problems globally [1, 2], in both high-income as well as low- and middle-income countries [3]. However, awareness about the disease and its prevention and treatment are low in low- and middle-income countries compared to developed countries [3]. According to the Global Burden of Disease Study, hypertensive heart disease accounts for 17.5 million disability-adjusted life years in 2015 [4]. Optimal control of blood pressure is paramount to prevent hypertension-related complications and deaths [5]. High adherence to antihypertensive medications can effectively lower hypertension-related complications and improve survival [6, 7].

Medication adherence is defined as “the extent to which the medication-taking behavior of a patient corresponds with agreed recommendations/prescriptions.” Patients receiving medication need to understand that the medications are critical to achieving blood pressure control [8, 9]. Many studies have suggested that a high level of adherence to antihypertensive drug treatment is related to better blood pressure (BP) control and a reduced risk of cardiovascular disease (CVD) [8, 10–13]; however, low adherence to antihypertensive drugs is a major public health and clinical challenges in the treatment of hypertension in low- and middle-income countries [14, 15]. Patients who poorly adhere to antihypertensive medications have a higher risk of adverse outcomes, including hospitalization, and incur higher healthcare costs due to complications as compared to patients who had good adherence [12, 13, 16, 17].

Multiple factors including patients' beliefs about health, illness, and treatment contribute to antihypertensive medication adherence therapy [18–23]. In treating hypertension, understanding patient's beliefs about medication adherence is fundamental because hypertension is silent and asymptomatic. Thus, patients might have misperceptions about hypertension, its severity, and the significance of its management [24, 25]. Socioeconomic status (poverty), low level of education, unemployment, lack of effective family/social support, and forgetfulness are also associated with adherence [26]. The patients' illness representations also have a direct influence on adherence to treatment [27].

Although the importance of treatment adherence has been recognized in Ethiopia by prior studies, the previous studies were conducted on a small number of patients often drawn from a single hospital and gave emphasis mostly only to sociodemographic factors [28–32]. Important independent factors that could influence patients' adherence to their treatment such as illness perception and health belief were hardly studied in Ethiopia. Hence, this study assessed the magnitude of antihypertensive treatment adherence and factors associated with it in multiple hospitals in Central Ethiopia.

## 2. Material and Methods

### 2.1. Study Design and Setting

This is a hospital-based cross-sectional study conducted from December 4, 2016, to September 25, 2017. The study was conducted in 4 public hospitals, namely, Bishoftu, Adama, Asella, and Shashemene. These hospitals are located in Oromia Regional State in Central Ethiopia. The hospitals provide a comprehensive healthcare service for both outpatient and inpatient clients. These hospitals also have specialty clinics where patients with specific chronic diseases are referred for follow-up. The hypertension clinic is one of those clinics. In Ethiopia, prescriptions are written by a physician in the follow-up clinics and patients get their medications from pharmacies from the same facility or pharmacies outside the healthcare facilities.

### 2.2. Study Population

All adults following their antihypertensive treatment in the four study hospitals were and who fulfilled the inclusion criteria were included in the study. The inclusion criteria include age at least 18 years or above, a diagnosis of hypertension confirmed by a physician, patients on antihypertensive medication for at least 3 months, patients who can give consent to participate in the study, and patients with no acute distress related to any disease during recruitment of study participants. Pregnant women, patients who cannot give consent, and patients who have hearing and/or speaking problems were excluded from the study.

### 2.3. Sample Size and Sampling Procedures

The sample size for determining the proportion of adherence to hypertensive treatment was determined with the following assumption: adherence to antihypertension treatment taken as 64.6% based on Ambaw et al. study [28], a 4% precision, 95% level of confidence, and 10% nonresponse rate. Accordingly, the calculated sample size was 604. For factors associated with adherence to antihypertensive treatment, we used a research conducted by [28]. Using StatCalc, the most determinant factor for treatment adherence was residence of the patients and considering *P*_1_=28.7, OR = 0.64, 80% power, 95% confidence level, and 1 : 1 (*n*_1_ = *n*_2_) ratio. After adding 10% nonresponse, the calculated sample size was 1030 (515 urban and 515 rural). Hypertensive patients who consented for the study were consecutively enrolled until the required sample was fulfilled.

### 2.4. Data Collection

Data were collected using a uniform and pretested questionnaire by 12 trained nurses (3 for each hospital). The study nurses were not working at the hypertension clinic. Data were collected through a face-to-face interview. The data collectors were competent to do interviews either in Amharic (the national language) and Afaan Oromo (the state language). Additionally, one nurse was assigned to supervise the data collection process at each hospital. Patients were interviewed after they got their routine services at existing. Before collecting data, the data collectors explained the objective of the study and obtained informed verbal consent from each study participant. The data collection tool contained questions on sociodemographic information, lifestyle, health-related matters, illness perception (IPQ-R) [33], the Morisky Medication Adherence Scale (MMAS-8) [34], and health beliefs. Some of these components are described in more detail below.

### 2.5. Illness Perception Questionnaire-Revised (IPQ-R)

The Illness Representation (IR) component of the validated IPQ-R [33] had seven subscales and 34 questions including the subdimensions of the timeline, timeline cyclical or symptoms fluctuate over time, consequences, personal control, treatment control, illness coherence, and emotional representations. In all dimension's subjects were given 5 options which were converted to a 5-point Likert-type scale for result analysis: strongly disagree (1), disagree (2), neither agree nor disagree (3), agree (4), and strongly agree (5).

For all these subscales, partial scores were defined as the mean of the scores for the items on each subscale (considering direct and inverse items, eight negative item scores were reversed: 1, 6, 13, 15, 19, 22, 23, and 32). Then, responses of all items are summed according to the IR dimensions (4 items of the timeline, 4 items of timeline cyclical, 6 items of consequences, 4 items of personal control, 5 items of treatment control, 5 items of illness coherence, and 6 items of emotional perceptions) and then dichotomized in to high and low by their mean. A higher score indicates stronger beliefs about the disease chronicity, cyclical course, impact and outcomes, personal influence, cure possibilities, perceived understanding, and emotional reactions to the disease. The internal consistency of Cronbach's alpha of the IPQ-R was checked and was 0.78, indicating satisfactory internal consistency.

### 2.6. Hypertension Belief and Behavior Questionnaire

The questionnaire is generated by referring to previously published studies based on the constructs of the HBM [35]. The HBM consisted of perceived susceptibility, perceived severity, perceived benefits, perceived barriers, perceived self-efficacy, and cues to action. The scores of each one of the HBM constructs were evaluated based on a 5-item Likert scale. Perceived susceptibility, perceived severity, perceived benefits, perceived barriers, perceived self-efficacy, and cues to action consisted of 4, 5, 5, 5, 4, and 4 questions, respectively.

### 2.7. Adherence to Medication

Medication adherence was measured using a validated eight-item self-reported Morisky Medication Adherence Scale (MMAS-8) [34]. Each item measures specific medication-taking behavior. Approval was obtained from professor Morisky to use his scale. The questions are phrased to avoid the “yes-saying” bias by reversing the wording of the questions about the way patients might experience failure in following their medication regimen since there is a tendency for patients to give their physicians or other healthcare providers positive answers [34].

This scale is a questionnaire with high reliability and validity, which has been particularly useful in chronic conditions such as hypertension. Response choices were “Yes” or “No” for items 1 through 7, and item 8 has a four-point Likert response scale. Total scores on the MMAS-8 ranged from 0 to 8, with scores of 8 reflecting high adherence, 6 to <8 reflecting medium adherence, and <6 reflecting low adherence. The internal consistency of Cronbach's alpha of the MMAS was checked and was 0.72 [34, 36, 37].

### 2.8. Statistical Analyses

Descriptive statistics were used to summarize sociodemographic, disease characteristics of the study population, and the nature and frequency of antihypertensive medications used. We examined sociodemographic, health-related variables, illness representation variables, and health belief items considering adherence as an outcome variable. The variables which have a *P*. value of 0.25 in bivariate analysis entered into multivariable ordinal logistic regression analysis. The ordinal logistic regression model was used because the outcome variable had three ordered levels of adherence (low⁄ medium/high). This model compared adjacent levels: the odds ratio (OR) corresponded with the odds of adherence to the next lower level. The ORs are presented with their 95% confidence intervals (CIs). The level of significance was set at a *P* value of less than 0.05. STATA 12 software was used for data entry and analyses.

## 3. Result

A total of 989 study participants were interviewed from four public hospitals in Central Ethiopia. The response rate was 96.0%. The mean age of respondents was 57.6 years (SD = 11.8). Orthodox Christians and the Oromo ethnic group accounted for 64.6% and 51% of the respondents, respectively. More than three-fourth (76.8%) of the respondents were urban residents. Six hundred twenty-seven (63.4%) reported to be married, 34.6% were illiterate, and 32.9% were housewives (Table 1).

About three-fourth (73.8%) of the respondents reported that it takes half an hour or more for a single trip to reach the hospital where they are receiving antihypertensive treatment. More than half (55.7%) of the respondents visit the hospital once every month; about three-fourth (72.7%) did not have a family history of hypertension, and the majority (79.1%) reported getting support from their family or friends (Table 2).

Concerning health-related conditions of the respondents, 42.8% were on treatment for about one to five years, 51.3% took two drugs daily, and about half (51.8%) took their drugs more than once per day. Three-fourth (75.1%) of the respondents reported no side effects of medications, 70.7% reported never heard about the side effects of the drug from healthcare providers, only 38.2% reported the cost of the drug is high, and the rest reported either fair or low. About half (52.9%) of the respondents had comorbid chronic illnesses, and the most frequently reported comorbidity was diabetes mellitus by 80.1% (*n* = 523). More than three-forth (81.3%) of the respondents sleep for more than 6 hours with an average of 6.95 hours (SD = 1.98). The mean values of systolic blood pressure were 139.56 (SD = 15.9) mmHg and 86.3 (SD = 8.32) mmHg for diastolic blood pressure (Table 3).

Concerning the respondent's illness representation, the mean scores of the seven dimensions of illness representation were 13.2 ± 1.6 for timeline, 13.2 ± 2.2 for coherence, 13.1 ± 1.9 for controllability-personal, 14.5 ± 2.6 for controllability-treatment, 20.4 ± 2.8 for a consequence, 11.1 ± 3.1 for timeline cyclical, and 17.9 ± 4.3 for emotional representation, respectively. This information indicates that patients believed that hypertension tended to be relatively chronic, predictable, and controllable through personal effort or by medical treatment and that hypertension had relatively little influence on their lives. Patients also believed that they had a relatively good understanding of their hypertension and rarely felt negative emotions due to hypertension (Table 4).

In this study, the researcher tried to assess hypertensive patient's beliefs using the health belief constructs. The findings indicated that respondents had lower beliefs of risk to contracting an illness or its complications (perceived susceptibility), lower beliefs that potential factors might make it difficult to take the behavior (perceived barriers), and lower internal or external cues that prompt the action, whereas they had higher beliefs that the disease is severe and has serious consequences (perceived severity), higher beliefs that taking treatment is beneficial to reduce or prevent disease threat (perceived benefit), and relatively good confidence to tackle their illness (self-efficacy) (Table 5).

Considering the frequency distribution of the MMAS-8 items, the majority or more than three-fourth of the respondents reported positive responses for MMAS-8 items. However, MMAS-8 items sometimes forget taking their drugs and having some degree of remembering problem to take their medications reported less than three-fourth of the respondents, 63.3% and 52.3%, respectively (Table 6).

Use of the ©MMAS is protected by US copyright and registered trademark laws. Permission for use is required. A license agreement is available from MMAR, LLC., Donald E. Morisky, 294 Lindura Court, Las Vegas, NV 89138-4632, dmorisky@gmail.com.

**Figure 1:**
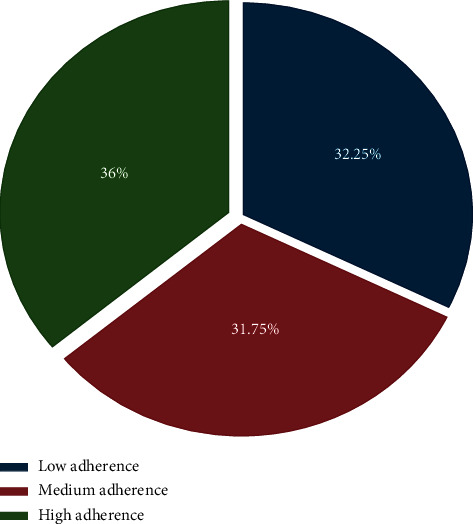
Hypertensive patient's level of treatment adherence in Central Ethiopia, 2017.

Overall, 32.3% (95% CI: 29.1, 35.0) of participants demonstrated low adherence, 31.7% (95% CI: 29.0, 34.7) medium adherence, and 36.0% (95% CI: 33.0, 38.9) high adherence. About two-third (67.7%) (95% CI: 64.7, 70.7) of participants were either moderate or high adherent for their antihypertensive medications removed (Figure 1).

In bivariate regression analysis, sex, educational status, occupation, frequency of visit, family support, perception on a timeline, perception on a consequence, cyclical perception, perceived susceptibility, perceived benefit, perceived severity, and cues to action had a significant association with treatment adherence.

After controlling possible confounding effects of other covariates, six factors remained as significant independent predictors of treatment adherence in the adjusted ordinal logistic regression model. These are farmer (AOR: 0.51; 95% CI = 0.33, 0.79), family support (AOR: 1.6; 95% CI = 1.23, 2.22), perceived consequences of hypertension (AOR: 1.51; 95% CI = 1.17, 1.95), perceived cyclical nature of hypertension (AOR: 0.76; 95% CI = 0.58, 0.95), perceived susceptibility (AOR: 0.61; 95% CI = 0.48, 0.78), and perceived severity of the disease (AOR: 1.42; 95% CI = 1.09, 1.86) had significant association with treatment adherence (Table 7).

## 4. Discussion

This study found that only about one-third of the respondents had a high level of treatment adherence. Being a farmer, having family support, higher perceived consequences, perceived cyclical nature of hypertension, perceived susceptibility, and perceived severity of the disease had a significant association with treatment adherence.

We collected the data for this study using an interviewer-administered questionnaire, which is a preferred method when conducting a fairly large survey among the community with substantial low literacy levels, to minimize cognitive burden and increase response rate [38]. To minimize social desirability bias to self-reported adherence, we used a standard questionnaire and carefully selected and trained data collectors to conduct the interviews in a nonjudgmental manner. Thus, the adherence level reported in this study is a fair reflection of the practice in the study areas.

In our study, higher adherence to hypertensive medication as measured by MMAS-8 was only 36.0%, which is comparable to several reports from low- and middle-income countries [39–43]. However, it is lower than some other studies in Ethiopia such as that reported from Gondar hospital, 64.6% [28], Jimma Hospital, 55.7% [30], in selected hospitals of Addis Ababa, 66.8% [32], and Tikur Anbessa hospital in Addis Ababa, 69.2% [31]. The possible reasons for this difference could be variations in the population groups and cutoff points to adherence between the studies. Another explanation might be due to differences in the study population characteristics, differences in sample size, and the area covered by the research. The present study covered larger areas compared to the above studies. This low level of adherence can be averted by providing patients with meaningful information or health education about treatment adherence.

The findings of this research revealed that farmers were less likely to adhere to antihypertensive treatment compared to employed people. The Farmer is likely to be less educated and may have less information on the treatment adherence compared to the employed person [44, 45]. Therefore, healthcare providers should prepare health education to their clients based on their level of awareness by a due emphasis on farmers.

In this study, respondents who had family support were more likely to adhere to their treatment compared to those who have no family support. This finding is similar to the studies conducted in Northern Ethiopia, Mekele [29], and Nigeria [46], and an absence of household support had a strong negative effect on adherence among hypertensive patients. Likewise, studies in Congo reported that patients who received no support from family members about taking their medications were more likely to be noncompliant than the others [47]. The reason for this could be family support is important in the long-term management of hypertension, which requires a life-long change in the lifestyle of the affected person. Strong perceived family support will improve their self-worth and motivation. It is plausible that a motivated hypertensive patient will adhere to therapeutic plans and, therefore, achieve better blood pressure control.

While there may be no clear symptoms of hypertension, understanding how patients perceive hypertension (their illness perceptions) is necessary because, without this, treatment may not be appropriately tailored to their needs and belief systems. Understanding individuals' perceptions about their illnesses provide a key for developing effective strategies to cope with chronic illnesses. Perception of a lower ability to control a health threat can be a barrier to one's behavioral actions, or cognitive and emotional changes in illness representations because of somatic experiences. Higher perception of illness control is associated with lower anxiety, lower avoidance/denial of coping strategies, and positive reappraisal [48, 49]. In the present study, among illness perception dimensions perceived consequences and cyclical perception had a significant association with treatment adherence.

The respondent's perception of the consequences of the disease was also positively related to treatment adherence, which was consistent with reports by other researchers [50, 51]. The perception of consequences has been shown to improve motivation to adhere to antihypertensive treatment [52].

Patients' beliefs about how long their symptoms will last might impact their decisions to seek or not seek healthcare. Cyclical timeline refers to the belief of an individual that the expected symptoms will diminish after a certain period and then they might reappear at a certain time in the future. When the symptoms diminished, patients believe that it is cured and more likely to be nonadherent, including decreasing frequency and types of medication, taking medications intermittently, and deviating from the prescribed timing. In this regard, respondents who had higher cyclical perception were less likely to adhere to their treatment. The findings of this research were also supported by a study conducted by Chen et al. [49] indicated that a belief that the illness (hypertension) cyclical was associated with nonadherence to treatment.

In treating hypertension, understanding patient's beliefs about medication adherence is fundamental because hypertension is often silent and asymptomatic. Thus, patients might undermine its severity and the significance of its management [27, 30, 53], leading to poor adherence to antihypertensive medication.

According to the Health Belief Model (HBM) people's perception of risk predicts their behaviors, low-risk perception leads to undesired behaviors [52]. Hypertensive patients with low-risk perception may have a risky lifestyle and poor adherence to prescribed medications, which significantly compromise the control of the condition [54, 55].

Under the constructs of the HBM, those with a higher perceived susceptibility showed better adherence to antihypertensive medications [55, 56]. A study conducted among patients in South India found that medication adherence was significantly associated with all the six components of the HBM [57], and a Chinese study found that higher levels of perceived susceptibility, cues to action, and self-efficacy and a lower level of perceived barriers were significantly associated with better antihypertensive medication adherence [58]. However, in our study, only perceived susceptibility and perceived severity to hypertension showed a significant association with treatment adherence.

The importance of understanding treatment adherence to antihypertension medications in controlling the patient's blood pressure and reducing hypertension-related complications to clinical practice cannot be overemphasized. Healthcare providers have to be aware of and understand patient's beliefs about their illness and medications while caring for them and incorporating this belief in designing effective interventions to improve medication adherence through reducing barriers to taking medications.

Overall, 67.7% of patients were reporting a medium or high level of adherence to antihypertensive medication in this research. Among the study participants, farmers by occupation, having family support, perceived consequences, perceived cyclical nature of hypertension, perceived susceptibility, and perceived severity of the disease showed a significant association with treatment adherence. Illness perceptions and other beliefs may influence the actions of the individual and negatively or positively affect his or her health and taking medication. Therefore, it is important before and during treatment to assess patients' views about their illness, symptoms, and treatments.

## Figures and Tables

**Table 1:** Sociodemographic characteristics of hypertensive patients in Central Ethiopia, 2017 (*n* = 989).

Variable	Frequency	%
Sex		
Male	468	47.3
Female	521	52.7
Name of the hospital		
Asella	322	32.5
Adama	268	27.1
Bishoftu	246	24.9
Shashemen	153	15.5
Age		
20–35	43	4.3
35–49	241	24.4
50–64	512	51.8
65–79	193	19.5
Mean and SD (57.6 ± 11.8)		
Religion		
Orthodox	639	64.6
Muslim	221	22.4
Protestant	129	13.0
Address		
Urban	760	76.8
Rural	229	23.2
Level of education		
Illiterate	342	34.6
Read and write	148	14.9
Primary	177	17.9
Secondary	172	17.4
Diploma and above	150	15.2
Marital status		
Single	154	15.6
Married	627	63.4
Divorced	55	5.5
Widowed	153	15.5
Ethnicity		
Oromo	504	50.9
Amhara	377	38.1
Tigre	26	2.6
Gurage	52	5.3
Others	30	3.1
Occupation		
GOV employed	154	15.6
Self-employed	151	15.3
Farmer	129	13.0
Housewife	325	32.9
Retired	230	23.2

**Table 2:** Health-seeking and illness-related factors of hypertensive patients in Central Ethiopia, 2017 (*n* = 989).

Variable	Frequency	%
Frequency of visit		
Once or less per month	633	64.0
Once in 2 months or more	356	36.0
Distance of residence		
≤30 minutes	259	26.19
>30 minutes to <1 hour	379	38.32
≥1 hour	351	35.49
Family history of hypertension		
Yes	234	23.7
No	755	76.3
Presence of family support		
Yes	782	79.1
No	207	20.9

**Table 3:** Health-related conditions of hypertensive patients in Central Ethiopia, 2017 (*n* = 989).

Variable	Frequency	%
Duration on antihypertensive treatment		
<5 years	538	54.4
≥5 years	451	45.6
Number of antihypertensive drugs taken		
One	429	43.4
More than one	560	56.6
Antihypertensive drugs dosage/frequency		
Once per day	477	48.2
More than once	512	51.8
Any history of side effect to antihypertensive drugs		
Yes	246	24.9
No	743	75.1
Ever told about the side effect of antihypertensive drugs		
Yes	290	29.3
No	699	70.7
Perceived cost of the antihypertensive drugs		
Low	113	11.4
Fair	498	50.4
High	378	38.2
Anything that makes you stressed		
Yes	523	52.9
No	466	47.1
Duration of sleep in an hour		
<4 hours	55	5.5
4 to 8 hours	787	79.6
>8 hours	147	14.9
Level of BP		
Controlled	415	41.9
Uncontrolled	574	58.1

**Table 4:** Illness perception of hypertensive patients in Central Ethiopia, 2017 (*n* = 989).

Variable	Frequency	%
Timeline acute/chronic		
Low	469	47.4
High	520	52.6
Consequence		
Low	460	45.5
High	529	53.5
Treatment control		
Low	428	43.28
High	561	56.72
Personal control		
Low	422	43.3
High	567	56.7
Illness coherence		
Low	544	55.1
High	445	44.9
Timeline cyclical		
Low	495	50.1
High	494	49.9
Emotional representation		
Low	523	52.9
High	466	47.1
Illness representation		
Low	483	48.8
High	506	51.2

**Table 5:** Hypertension belief and behavior of patients in Central Ethiopia, 2017 (*n* = 989).

Variable	Frequency	%
Perceived susceptibility		
Lower	588	59.5
Higher	401	40.5
Perceived severity		
Lower	339	34.3
Higher	650	65.7
Perceived benefits		
Lower	226	22.9
Higher	763	77.2
Perceived barriers		
Lower	518	52.4
Higher	471	47.6
Cues to action		
Lower	563	56.9
Higher	426	43.1
Self-efficacy		
Lower	478	48.3
Higher	511	51.7

**Table 6:** Responses of hypertensive patients to MMAS-8 questions in Central Ethiopia, 2017 (*n* = 989).

Variable	Frequency	%
Sometimes forget taking their drugs		
Yes	363	36.7
No	626	63.3
Had a problem taking in the last 2 weeks		
Yes	164	16.6
No	825	83.4
Stop taking or decrease the dose		
Yes	130	13.1
No	859	86.9
Forget to bring along when traveling		
Yes	210	21.2
No	779	78.8
Took their medication yesterday		
Yes	807	81.6
No	182	18.4
Stop taking when they feel controlled		
Yes	149	15.1
No	840	84.9
Felt hassled sticking treatment plan		
Yes	156	15.8
No	833	84.1
Some degree of remembering a problem		
Almost never	517	52.3
Sometimes	408	41.2
Frequently	56	5.7
Always	8	0.8

**Table 7:** Effect of selected variable and other characteristics on adherence to antihypertensive treatment in Central Ethiopia, 2017.

Variable	Treatment adherence			COR (95% CI)	AOR (95% CI)	*P* value
	Low	Medium	High			
Sex						
Male	166	144	158	1	1	
Female	153	170	198	1.26 (0.99, 1.58)	1.21 (0.94, 1.55)	0.133
Educational status						
Illiterate	98	113	131	1.57 (1.10, 2.24)^*∗*^	1.39 (0.94, 2.06)	0.103
Read and write	52	42	148	1.31 (0.86, 1.99)	1.30 (0.83, 2.03)	0.245
Primary	55	56	177	1.46 (0.97, 2.18)	1.28 (0.83, 1.95)	0.261
Secondary	54	57	172	1.39 (0.93, 2.08)	1.26 (0.82, 1.92)	0.290
Diploma and above	60	46	150	1	1	
Occupation						
Gov. employed	46	38	70	1	1	
Self-employed	44	44	63	0.92 (0.60, 1.40)	0.86 (0.56, 1.32)	0.481
Farmer	54	41	34	0.49 (0.32, 0.76)^*∗*^	**0.51 (0.33, 0.79)** ^*∗*^	**0.003**
House wife	108	108	109	0.69 (0.49, 0.99)^*∗*^	0.71 (0.49, 1.02)	0.068
Retired	67	83	80	0.78 (0.53, 1.14)	0.70 (0.48, 1.05)	0.084
Frequency of visit						
Once in a month	159	180	212	1	1	
>Once in month	27	25	30	1.74 (1.03, 1.76)^*∗*^	1.04 (0.67, 1.61)	0.856
Once in 2 months	42	17	33	1.17 (0.75, 1.86)	0.73 (0.47, 1.14)	0.164
Once in 3 months or more	91	92	81	0.85 (0.54, 1.34)	0.92 (0.69, 1.22)	0.565
Family support						
Yes	235	252	295	**1.52 (1.14, 2.02)** ^*∗*^	**1.65 (1.23, 2.22)** ^*∗*^	**0.001**
No	84	62	61	**1**	1	
Treatment control						
Lower	141	148	133	1	1	
Higher	178	166	233	0.81 (0.63, 1.02)^*∗*^	0.86 (0.67, 1.11)	0.246
Perceived consequence						
Lower	162	156	142	1	**1**	
Higher	157	158	214	1.18 (1.17, 1.88)^*∗*^	**1.51 (1.17, 1.95)** ^*∗∗*^	**0.001**
Cyclical perception						
Lower	162	173	209	1	**1**	
Higher	157	141	147	0.77 (0.61, 0.98)^*∗*^	**0.76 (0.58, 0.95)** ^*∗*^	**0.019**
Perceived susceptibility						
Lower	172	165	251	1	**1**	
Higher	147	149	105	0.59 (0.46, 074)	**0.61 (0.48, 0.78)** ^*∗*^	**0.0001**
Perceived severity						
Lower	138	105	96	1	**1**	
Higher	181	209	260	1.73 (1.36, 2.21)^*∗*^	**1.42 (1.09, 1.86)** ^*∗*^	**0.010**
Perceived benefit						
Low	76	78	72	1	1	
High	243	236	284	1.17 (0.89, 1.54)	1.09 (0.82, 1.46)	0.547
Cues to action						
Low	171	184	208	1	1	
High	148	130	148	0.86 (0.69, 1.09)	0.89 (0.69, 1.115)	0.373

## Data Availability

The datasets generated and/or analyzed during the current study are not publicly available due to some privacy reasons, but part of the raw dataset will be available from the corresponding author upon reasonable request.
